# Supplementary material for: Factors Associated with Antimicrobial Stewardship Practices on California Dairies: One Year Post Senate Bill 27
Source: Antibiotics (Basel). 2022 Jan 27;11(2):165. doi: 10.3390/antibiotics11020165 (PMC8868138; doi:10.3390/antibiotics11020165)
Supplement: Supplementary file 1 [file antibiotics-11-00165-s001.zip › Antibiotics Table S1_012622.pdf]

Table S1. Summary of herd characteristics from 113 responses received from conventional dairies to a mailed questionnaire on antimicrobial drug (AMD) use in adult cows on California during 2019.

			95% Confidence limits	
Variables	n	Estimate (%)	Lower	Upper
**Respondent role**	**113**			
Manager	23	20.35	13.83	28.92
Owner	66	58.41	48.99	67.24
Owner-Manager	21	15.58	12.36	26.97
Veterinarian	3	2.65	0.84	8.03
**Region**	**113**			
Northern California (NCA)	8	7.07	3.54	13.65
Northern Central (NSJV)	53	46.92	37.77	56.24
Greater southern California (GSCA)	52	46.01	36.92	55.37
**Herd size (# of cow)**	**113**			
≤1304	61	53.98	44.62	63.07
>1304	52	46.01	36.92	55.37
**Rolling herd average (kg/cow)**	**107**			
<10,880	19	17.76	11.54	26.32
≥10,880	88	82.24	73.67	88.46
**Bulk tank somatic cell count (cells/mL)**	**112**			
<100,000	14	12.50	7.48	20.14
100,000-199,999	69	61.61	52.15	70.25
≥200,000	29	25.89	18.52	34.92
**Breed**	**112**			
Holstein	63	56.25	46.81	65.25
Jersey	3	2.68	0.85	8.11
Crossbred	6	5.36	2.39	11.54
Mixed herds (Holstein and Jersey)	40	35.71	27.28	45.13
