# Supplementary material for: Factors Associated with Antimicrobial Stewardship Practices on California Dairies: One Year Post Senate Bill 27
Source: Antibiotics (Basel). 2022 Jan 27;11(2):165. doi: 10.3390/antibiotics11020165 (PMC8868138; doi:10.3390/antibiotics11020165)
Supplement: Supplementary file 1 [file antibiotics-11-00165-s001.zip › Antibiotics Table S2_012622.pdf]

Table S2: Estimated coefficients and odds ratios from a multilevel mixed effects logistic regression model for the association between survey factors and decreased use of antimicrobial drugs (AMD) that were previously available over the counter.

Variables	Coefficient	SE	Odds Ratio	95% CI		
				Lower	Upper	*P*-value[1]
**Region[2]**						
GSCA	Referent					
NCA + NSJV	0.42	0.53	1.53	0.53	4.37	0.43
**Herd size**						
<1304	Referent					
≥1304	1.83	0.68	6.30	1.64	24.10	<0.01
**Breed**						
Holstein	Referent					
Jersey	0.21	1.76	1.23	0.03	39.66	0.91
Crossbred	1.25	1.46	3.52	0.20	61.91	0.39
Mix/Other	1.01	0.68	2.75	0.71	10.58	0.13
**Use or increased use of preventive alternatives to AMD since 2018**						
No	Referent					
Yes	1.17	0.58	3.23	1.02	10.21	0.04

[1]Factors are statistically significant at $P \leq 0.05$.

[2] Northern California (NCA), Northern San Joaquin Valley (NSJV), and Greater Southern California (GSCA)
