# Supplementary material for: Factors Associated with Antimicrobial Stewardship Practices on California Dairies: One Year Post Senate Bill 27
Source: Antibiotics (Basel). 2022 Jan 27;11(2):165. doi: 10.3390/antibiotics11020165 (PMC8868138; doi:10.3390/antibiotics11020165)
Supplement: Supplementary file 1 [file antibiotics-11-00165-s001.zip › Antibiotics Table S3_012622.pdf]

Table S3: Estimated coefficients and odds ratios from a multilevel mixed effects logistic regression model for the association between survey factors and having farm made changes in management to prevent spread of disease.

Variables	Coefficient	SE	Odds Ratio	95% CI		*P*-value[1]
				Lower	Upper	
**Region[2]**						
GSCA	Referent					
NCA + NSJV	-1.20	0.72	0.30	0.07	1.22	0.09
**Herd size, milking cows**						
<1304	Referent					
≥1304	-0.70	0.74	0.49	0.11	2.14	0.35
**Breed[3]**						
Holstein	Referent					
Other (Jersey, crossbred, mix)	0.88	0.70	2.41	0.61	9.55	0.21
**Who review/revise the animal health protocols**						
Dairy personnel	Referent					
Veterinarian	2.60	1.10	13.49	1.71	106.47	0.01
**Farm's animal health**						
No Change	Referent					
Better	1.56	0.69	4.79	1.21	18.87	0.02
Worse	0.72	1.43	2.04	0.12	34.23	0.62
**Use or increased use of protective alternatives to AMD since 2018**						
No	Referent					
Yes	1.50	0.65	4.40	1.23	15.72	0.02

[1]Factors are statistically significant at $P \leq 0.05$.

[2] Northern California (NCA), Northern San Joaquin Valley (NSJV), and Greater Southern California (GSCA)

[3] Breed was categorized into two levels: Holstein and others (Jersey, crossbreed, and mixed herds). Dairies with jersey and crossbreed cows were all did not report change in management practices.
