# Supplementary material for: Factors Associated with Antimicrobial Stewardship Practices on California Dairies: One Year Post Senate Bill 27
Source: Antibiotics (Basel). 2022 Jan 27;11(2):165. doi: 10.3390/antibiotics11020165 (PMC8868138; doi:10.3390/antibiotics11020165)
Supplement: Supplementary file 1 [file antibiotics-11-00165-s001.zip › Antibiotics Table S4_012622.pdf]

Table S4: Estimated coefficients and odds ratios from a multilevel mixed effects logistic regression model for the association between survey factors and decreased AMD cost compared to 2018.

Variables	Coefficient	SE	Odds Ratio	95% CI		
				Lower	Upper	*P*-value[1]
**Region[2]**						
GSCA	Referent					
NCA + NSJV	0.51	0.55	1.67	0.61	4.86	0.35
**Herd size, milking cows**						
<1304	Referent					
≥1304	0.33	0.58	1.39	0.45	4.32	0.56
**Breed**						
Holstein	Referent					
Jersey	1.82	1.41	6.20	0.40	97.40	0.20
Crossbred	-1.38	1.34	0.25	0.01	3.43	0.30
Mix/Other	-0.71	0.65	0.48	0.14	1.75	0.27
**Farm's animal health**						
No Change+ Worse	Referent					
Better	1.70	0.55	5.48	1.85	16.17	<0.01
**Use or increased use of alternatives to AMD since 2018**						
No	Referent					
Yes	2.11	0.58	7.89	2.54	24.57	<0.01

[1] Factors are statistically significant at $P \leq 0.05$.

[2] Northern California (NCA), Northern San Joaquin Valley (NSJV), and Greater Southern California (GSCA).
