# Supplementary material for: Factors Associated with Antimicrobial Stewardship Practices on California Dairies: One Year Post Senate Bill 27
Source: Antibiotics (Basel). 2022 Jan 27;11(2):165. doi: 10.3390/antibiotics11020165 (PMC8868138; doi:10.3390/antibiotics11020165)
Supplement: Supplementary file 1 [file antibiotics-11-00165-s001.zip › Antibiotics Table S5_012622.pdf]

Table S5. Summary of descriptive analysis of dairy producers' respondents classified as having "limited-moderate knowledge" or "good to excellent knowledge" in antimicrobial stewardship practices (AMS) based on responses obtained from 113 conventional California dairies.

Variables	AMS Knowledge of dairy producers in CA				*P*-value
	Limited to moderate		Good to excellent		
	N	% ± SE	N	% ± SE	
**Region**					
Greater southern CA	7/51	13.72 ± 4.81	44/51	86.27 ± 4.81	<0.01
Northern Central CA	15/51	29.41 ± 6.38	36/51	70.58 ± 6.38	<0.01
Northern CA	1/7	14.28 ± 13.22	6/7	85.71 ± 13.22	<0.01
**Herd size (milking cows/herd)**					
<1305	(15/59)	25.42 ± 5.66	(44/59)	74.00 ± 5.66	<0.01
≥1305	(8/50)	16.00 ± 5.23	(42/50)	84.00 ± 5.23	<0.01
**Annual Rolling herd average (Kg/cow)**					
<10,888	6/19	31.57 ± 10.95	13/19	68.42 ± 10.95	0.02
≥10,888	12/85	14.11 ± 3.79	73/85	85.88 ± 3.79	<0.01
**Use written/computerized health protocol**					
No	3/19	15.78 ± 8.40	16/19	84.21 ± 8.40	<0.01
Yes	19/78	24.35 ± 4.86	59/78	75.64 ± 4.86	<0.01
**Have drug inventory log**					
No	14/63	22.22 ± 5.23	49/63	77.77 ± 5.23	<0.01
Yes	8/43	18.60 ± 5.93	35/43	81.39 ± 5.93	<0.01
**Track AMD withdrawal periods**					
No	11/35	31.42 ± 7.84	24/35	68.57 ± 7.84	<0.01
Yes	12/74	16.21 ± 4.28	62/74	83.78 ± 4.28	<0.01
**Vaccinate cows against mastitis**					
No	6/20	30.00 ± 10.24	14/20	70.00 ± 10.24	0.01
Yes	12/78	15.38 ± 4.10	66/78	84.61 ± 4.10	<0.01
**Familiarity with MIADs**					
Not familiar	16/37	43.24 ± 8.14	21/37	56.75 ± 8.14	0.245
Familiar	7/71	9.85 ± 3.53	64/71	90.14 ± 3.53	<0.01
**Aware that MIADs require prescription**					
No	1/4	25.00 ± 21.65	3/4	75.00 ± 21.65	0.16
Yes	22/103	21.35 ± 4.03	81/103	78.64 ± 4.03	<0.01
