# Supplementary material for: Factors Associated with Antimicrobial Stewardship Practices on California Dairies: One Year Post Senate Bill 27
Source: Antibiotics (Basel). 2022 Jan 27;11(2):165. doi: 10.3390/antibiotics11020165 (PMC8868138; doi:10.3390/antibiotics11020165)
Supplement: Supplementary file 1 [file antibiotics-11-00165-s001.zip › Antibiotics Table S6_012622.pdf]

Table S6. Performance metrics of 3 classification models used to study the association between good to excellent antimicrobial stewardship practices and survey responses in 113 conventional California dairies during 2019.

	Classification model		
	Decision tree	Random forest	Gradient boosting
Specificity	78.26%	78.26%	78.26%
Sensitivity/Recall	32.56%	46.51%	60.47%
Precision	84.85%	88.89%	73.55
F1 statistics	47.06%	61.07%	66.37%
Balanced Accuracy	55.41	62.38%	69.36%
Area under ROC Curve (AUC)	0.81	0.74	0.96
