# Supplementary material for: Factors Associated with Antimicrobial Stewardship Practices on California Dairies: One Year Post Senate Bill 27
Source: Antibiotics (Basel). 2022 Jan 27;11(2):165. doi: 10.3390/antibiotics11020165 (PMC8868138; doi:10.3390/antibiotics11020165)
Supplement: Supplementary file 1 [file antibiotics-11-00165-s001.zip › Antibiotics_Figure S1_012622.pdf]

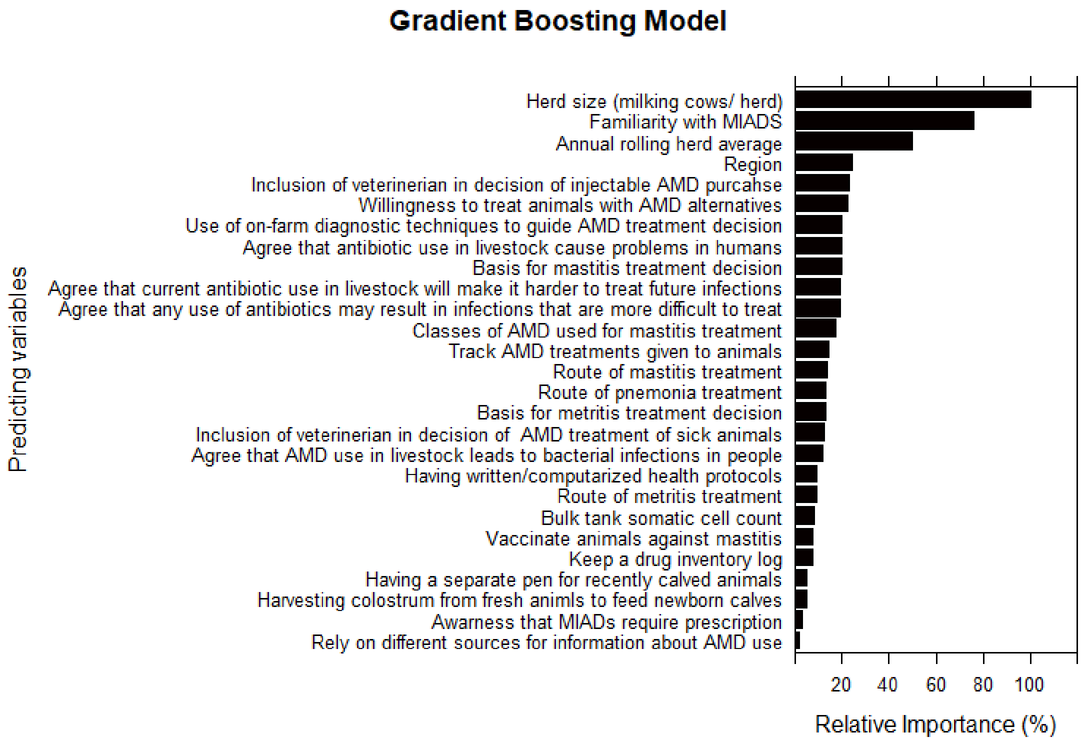

Figure S1. Predictive variables importance plot showing the ranking of variables relative importance for predicting dairies with good to excellent antimicrobial stewardship practices based on survey responses from 113 conventional California dairies during 2019. “MIADs” are medically important antimicrobial drugs. “AMD” is antimicrobial drug.
