# Supplementary material for: Factors Associated with Antimicrobial Stewardship Practices on California Dairies: One Year Post Senate Bill 27
Source: Antibiotics (Basel). 2022 Jan 27;11(2):165. doi: 10.3390/antibiotics11020165 (PMC8868138; doi:10.3390/antibiotics11020165)
Supplement: Supplementary file 1 [file antibiotics-11-00165-s001.zip › Antibiotics_Figure S2_012622.pdf]

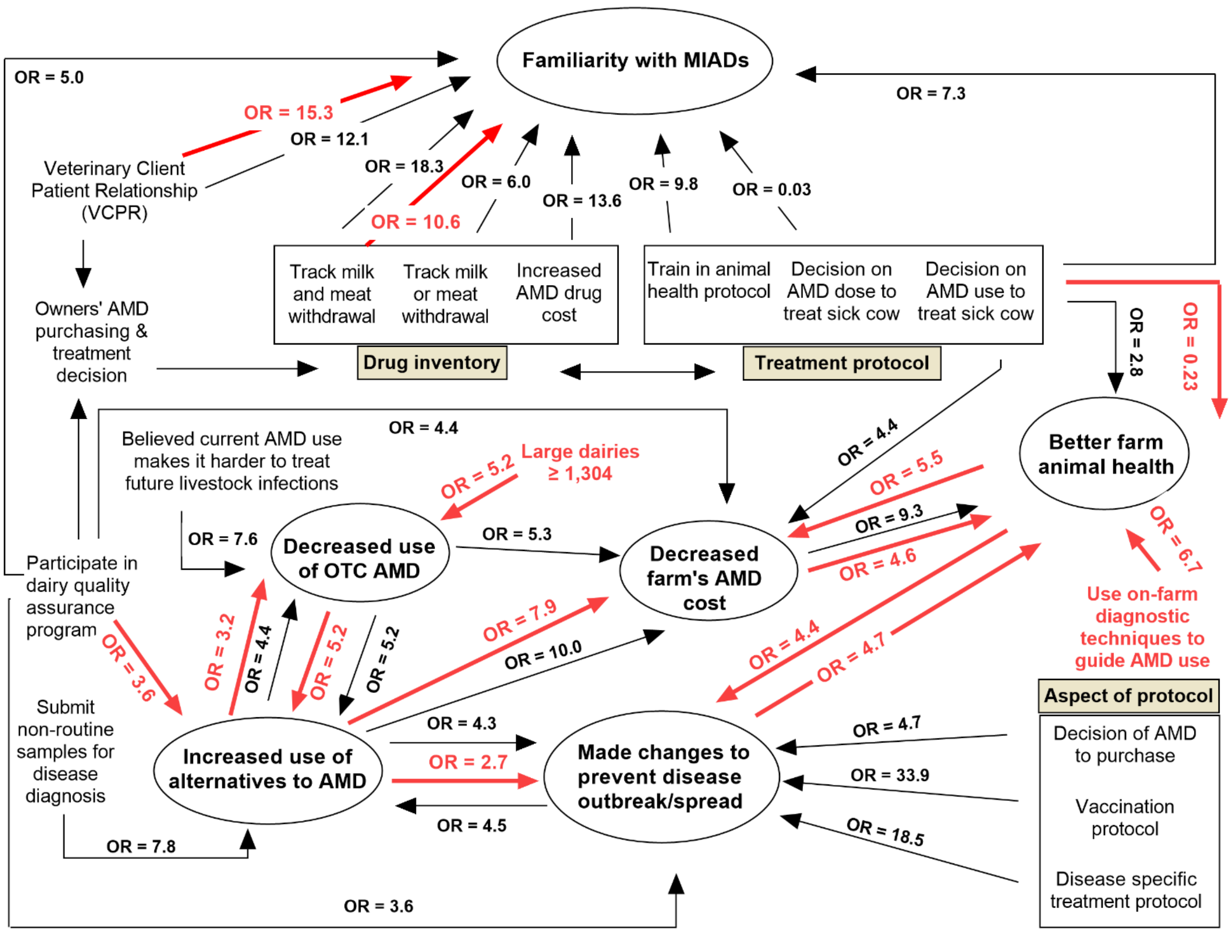

Figure S2. Web diagram showing the magnitude and direction of the associations between six antimicrobial stewardship outcomes and predictors from logistic regression models based on surveys of conventional California dairies during 2018 (black arrows), and 2019 (red arrows). Associations with both black and red arrows identify common associations between surveys.
